# Factors associated with glycemic control among diabetic adult out-patients in Northeast Ethiopia

**DOI:** 10.1186/s13104-018-3423-5

**Published:** 2018-05-18

**Authors:** Temesgen Fiseha, Ermiyas Alemayehu, Wongelawit Kassahun, Aderaw Adamu, Angesom Gebreweld

**Affiliations:** 1 Department of Clinical Laboratory Science, College of Medicine and Health Sciences, Wollo University, Dessie, Ethiopia; 2 Department of Medical Laboratory Science, College of Health & Medical Sciences, Debre Tabor University, Debre Tabor, Ethiopia

**Keywords:** Glycemic control, Fasting blood glucose, Diabetic mellitus, Northeast Ethiopia

## Abstract

**Objective:**

The aim of this study was to determine the status of glycemic control and identify factors associated with poor glycemic control among diabetic out-patients.

**Results:**

A hospital based cross-sectional study was conducted among randomly selected 384 (126 type 1 and 258 type 2) diabetic adults attending a hospital in Northeast Ethiopia from January 1 to April 30, 2017. Of the total participants, 70.8% had poor status of glycemic control (defined as mean fasting blood glucose level above 130 mg/dl). In the multivariate analysis, rural residence (AOR = 2.61, 95% CI 1.37–4.96), low educational level (AOR = 7.10, 95% CI 2.94–17.17) and longer duration of diabetes (AOR = 2.20, 95% CI 1.18–4.08) were significantly associated with increased odds of poor glycemic control. Moreover, merchants (AOR = 3.39, 95% CI 1.16–9.96) were significantly more likely to have poor glycemic control compared to government employee. Diabetic patients receiving oral anti-diabetics (AOR = 5.12, 95% CI 2.10–12.52) or insulin (AOR = 3.26, 95% CI 1.26–8.48) were more likely to be poorly controlled. These results highlight the needed for appropriate management of patients focusing on associated factors identified for poor glycemic control to maintain good glycemic control and improve adverse outcomes of the disease in this study setting.

## Introduction

Diabetes mellitus is a global public health problem, resulting in about 5 million deaths annually from related complications. It is estimated that more than 422 million adults are living with diabetes worldwide and this is expected to rich about 642 million by 2040 [1]. The burden of diabetes is highest in the developing world and mostly affects resources limited countries, where screening and access to care and treatment are not readily available [2]. Sub-Saharan Africa (SSA) is experiencing a significantly increasing prevalence of diabetes alongside other non-communicable diseases [3]. In 2015, 14.2 million adults were estimated to be living with diabetes in Africa, and this is projected to increase to 34.2 million by 2040 [1]. Moreover, nearly three-fourths of diabetes-related deaths occur in economically-productive people under the age of 60 years [1, 4]. As such, diabetes and associated complications could overwhelm health systems in SSA and leave many of those affected with substantial morbidity and mortality.

Poor and inadequate glycemic control constitutes a major public health problem and is a major risk factor for the development and progression of diabetes-related complications, which can greatly increase healthcare costs of the disease and reduce life expectancy and quality of life [5, 6]. Tightly controlling blood glucose is essential to diabetes care and management in order to delay the onset and decrease the incidence of complications [7]. Improved glycemic control has been shown to prevent the development and progression of diabetic complications [8, 9], as well as to increase the life expectancy and quality of life of patients [10]. Improved glycemic control has been also shown to significantly reduce diabetes related costs [11, 12]. Despite this evidence, a high proportion of patients with diabetes remains poorly controlled. This is particularly the case in most countries of SSA where the majority of patients in clinical care do not reach the optimal glucose targets [13, 14].

Ethiopia, like the rest of SSA countries, is experiencing an increasing prevalence of diabetes and the complications are now contributing a significant burden of disease in the country [15–18]. However, despite this growing prevalence of diabetes and is complications, data regarding glycemic control is scarce and little is known about the factors contributing for poor glycemic control. Such data are of great relevance for planning healthcare programs targeting improved diabetes control. The aim of this study was to determine the status of glycaemic control and identify factors associated with poor glycemic control among diabetic adults attending a hospital in Northeast Ethiopia.

## Main texts

### Methods

#### Study design, setting and data collection

A hospital based cross-sectional study was conducted at the out-patient diabetes clinic of Dessie Referral Hospital (DRH) in Northeast Ethiopia during the period from January to April 2017. DRH is found in Dessie town of Amhara region, located 401 km northeast of the capital of Ethiopia, and it serves as a referral center for the Wollo and surrounding zones. The hospital registers and treats all diagnosed diabetic patients and provides primary diabetes patient care in the region. During their visit participants were interviewed for collecting socio-demographic and risk factor variables by using a pre-tested structured questionnaire. Weight, height and blood pressure were measured at the time of the clinical examination performed and recorded by using a structured format. Body mass index (BMI) was calculated from weight (kg) in light clothing without shoes, and height (meters) without shoes. Blood pressure (BP) was measured in the right upper arm in the sitting posture, after a 5 min rest and three measurements were averaged to be recorded.

#### Participant eligibility criteria

For the purpose of this study, adult patients (≥ 18 years) with regular follow up and at least four measurements of fasting blood glucose (FBG) level were included. Based on the inclusion criteria, 384 randomly selected patients were included in the study by considering confidence level of 95% with margin of error 5% and prevalence rate of glycemic control 64.7% [19], and by adding 10% non-response rate. Patients were excluded if they were hospitalized and/or with critical illness or psychiatric disorders that rendered them unable to answer the questionnaires. Patients who did not provide consent to participate were also excluded from the study.

#### Measures and operational definitions

Clinical measures including duration of disease, type of diabetes and measurements of FBG level were abstracted from patients’ database. Participants FBG reading for at least 4 months were recorded for computing the mean blood glucose level, and poor glycemic control was operationally defined if FBG level was above 130 mg/dl. Participants, with regard to their smoking habit, were categorized as nonsmokers, if they had never smoked or quit smoking just a year before; and current smokers, if they smoke at least one cigarette for the last 12 months. Alcohol consumption was assessed by asking participant to report frequency of alcohol intake, accordingly at least twice weekly of any alcoholic drinks consumed was considered as alcohol consumer for the purpose of this analysis. Based on BMI, participants were grouped into different categories as normal (BMI < 25 kg/m^2^), overweight (BMI = 25–29.9 kg/m^2^) and obese (BMI ≥ 30 kg/m^2^). Hypertension was defined as systolic BP ≥ 140 mmHg or diastolic BP ≥ 90 mmHg or use of antihypertensive medication irrespective of the current BP.

#### Statistical analysis

Statistical analyses were carried out using SPSS version 20.0 software (SPSS Inc., Chicago, IL, USA). Data were expressed as mean ± standard deviation (SD) or percentage. Chi square (x^2^) test was used to compare proportions. Multivariate logistic regression was conducted and the corresponding adjusted odds ratios (AOR) and 95% confidence intervals (CI) were used to identify factors independently associated with poor glycemic control. *P* < 0.05 was used to indicate statistical significance.

#### Ethical consideration

Study protocol was approved by the Institutional Review Board of College of Medicine and Health Sciences, Wollo University. Official letter of cooperation was written to DRH, and permission from diabetic clinic of the hospital, where the data collection took place was obtained. Patient’s written informed consent to participate in the study was obtained after comprehensive explanation of the purpose and procedure of the study. Patients were informed about their rights to refuse or withdraw, and about confidentiality of the individual information obtained.

### Results

#### Socio-demographic and clinical characteristics of participants

This study included 384 participants, of which 52.3% were males. The mean (± SD) age of participants was 45 ± 14.5 years, ranging from 18 to 90 years and 78.4% of them were < 60 years old. The majority (70.3%) of participants were urban residents and more than half (54.4%) of the participants were married. About 31.3% participants were illiterate and 32% were housewives. More than two-third (71.1%) of the respondents had monthly income ≤ 1000 ETB (Table 1).

**Table 1:** Socio-demographic characteristics of study participants attending diabetic follow-up clinic in DRH, Northeast Ethiopia, 2017

Characteristics (N = 384)	Categories	n (%)
Age, mean ± SD		45 ± 14.5 years
Age group	18–39 years	151 (39.3)
	40–49 years	68 (17.7)
	50–99 years	82 (21.4)
	≥ 60–69 years	46 (12.0)
	≥ 70 years	37 (9.6)
Gender	Male	201 (52.3)
	Female	183 (47.7)
Residence	Rural	114 (29.7)
	Urban	270 (70.3)
Educational status	Illiterate	120 (31.3)
	Read and write	33 (8.6)
	1–8	95 (24.7)
	9–12	88 (22.9)
	College and above	48 (12.5)
Marital status	Single	79 (20.6)
	Married	209 (54.4)
	Divorced	44 (11.5)
	Widowed	52 (13.5)
Occupation	Students	41 (10.7)
	Employed	48 (12.5)
	Housewives	125 (32.6)
	Merchants	41 (10.7)
	Others	129 (33.6)
Monthly income	< 500 ETB	203 (52.9)
	500–1000 ETB	70 (18.2)
	> 1000 ETB	111 (29.9)

Table 2 shows clinical characteristics of the participants. Of the total participants, 67.2% were type 2 diabetic patients. The mean diabetes duration of participants was 6.87 ± 5.05 years and 28.6% had a duration of 10 or more years. Majority (98.7%) of the participants was nonsmokers/ex-smokers, 94.5% had no history of alcohol consumption, and 66.9% physically inactive. Mean BMI was 22.09 ± 5.60 kg/m^2^. The mean systolic and diastolic BP was 129.2 ± 13 and 82.4 ± 6 mmHg, respectively and 34.1% were hypertensive. Regarding diabetic medications, 61.7% of respondents were taking oral medication only followed by insulin (28.1%). The mean FBG level of the participants was 184.72 ± 89 mg/dl.

**Table 2:** Clinical characteristics of study participants attending diabetic follow-up clinic in DRH, Northeast Ethiopia, 2017

Characteristics (N = 384)	Category	n (%)
Type of diabetes	Type 1	126 (32.8)
	Type 2	258 (67.2)
Duration of DM	< 10 years	281 (73.2)
	≥ 10 years	103 (26.8)
Smoking status	Smokers	5 (1.3)
	Non-/ex-smokers	379 (98.7)
Alcohol drinking	Drinkers	21 (5.5)
	Non-drinkers	363 (94.5)
Physical activity		
Body mass index (kg/m^2^)	< 25 (normal weight)	321 (83.6)
	≥ 25 (overweight and obesity)	63 (16.4)
Hypertension	Yes	131 (34.1)
	No	253 (65.9)
Type of DM treatment	Oral anti-diabetics	239 (62.2)
	Insulin	106 (27.6)
	Oral anti-diabetics and insulin	39 (10.2)

#### Glycemic control

Of the total 384 participants, 112 (29.2%) had good glycemic control, while significant proportion of patients, 272 (70.8%) had poor glycemic control. Diabetes was more likely to be poorly controlled among rural residents (*P *= 0.002) and students or merchants (*P *= 0.015), and those with lower level of education (*P *< 0.001); longer duration of diabetes (*P *= 0.005); and hypertension (*P *= 0.008). Poor glycaemic control was significantly higher among participants on oral anti-diabetics or insulin than those on a combination of oral anti-diabetics and insulin (*P *< 0.001). Bivariate analysis of this study showed that factors such as age, gender, type of diabetes, monthly income, alcohol drinking, cigarette smoking, physical activity and BMI were not significantly associated with poor glycemic control.

Table 3 presents the multivariate analysis of factors associated with poor glycemic control. The rates of poor glycemic control was three times (AOR = 2.55, 95% CI 1.33–4.85) greater among rural than urban residents. The relative odds of poor glycemic control was seven (AOR = 7.10, 95% CI 2.94–17.17) and four times (AOR = 3.53, 95% CI 1.52–8.21) higher among participants who were illiterate and with informal education, respectively, than those with college/higher educational levels. Compared to those who had shorter duration of diabetes, participants who had longer duration of diabetes were 2-times (AOR = 2.20, 95% CI 1.18–4.08) more likely to have poor glycemic control. The odds of poor glycemic control was three times (AOR = 3.39, 95% CI 1.16–9.96) higher among merchants than government employee. Diabetic patients receiving only oral anti-diabetics and insulin were 5.2- and 3.4-fold more likely to have poor glycemic control, respectively (Table 3).

**Table 3:** Multivariate analysis of factors associated with poor glycemic control among diabetic out-patients attending DRH, 2017

Variables (n = 384)	Poor glycemic control, n (%)	Adjusted OR (95% CI)	P value
Residence			
Rural	91 (79.8)	2.61 (1.37–4.96)	0.004*
Urban	181 (67.0)	1	
Educational status			
Illiterate	96 (80.0)	7.10 (2.94–17.17)	< 0.001*
Read and write	25 (75.8)	3.53 (1.52–8.21)	0.003*
1–8	64 (67.4)	2.68 (0.92–7.80)	0.077
9–12	61 (69.3)	2.49 (0.69–8.99)	0.163
College and above	25 (52.1)	1	
Occupation			
Students	32 (78.1)	2.89 (0.99–8.00)	0.051
Employee	26 (54.2)	1	
Housewives	94 (75.2)	2.25 (0.81–6.24)	0.120
Merchants	33 (80.5)	3.39 (1.16–9.96)	0.026*
Others	86 (66.7)	0.95 (0.44–2.05)	0.895
Duration of DM (years)			
< 10	188 (66.9)	1	
≥10	84 (81.6)	2.20 (1.18–4.08)	0.013*
Type of treatment			
Oral anti-diabetics	185 (77.4)	5.13 (2.10–12.52)	< 0.001*
Insulin	70 (66.0)	3.26 (1.26–8.48)	0.015*
Oral anti-diabetics and insulin	17 (43.6)	1	

* Statistically significant at P value < 0.05

### Discussion

This study has revealed that more than two-third (70.8%) of diabetic adults in the study setting had poor glycemic control. The result of this study was comparable to those obtained in earlier studies that reported 70.9% [20] and 81.9% [21] of participants had poor glycemic control, but was higher than that of 64.7 and 59.4% [19, 22]. These results highlight the need to work more on optimum management of diabetes, as maintaining good glycemic control is main therapeutic goal for all patients with diabetes. The overall rates of poor glycemic control found in our study was higher than previous estimates from studies in Costa Rica (37%) [23], Zambia (61.3%) [24], and Kenya (60.5%) [25] but lower than that found in Uganda (73.5%) [13] and Brazil (76%) [26], which also included type 1 and 2 diabetic patients. This might be due to differences in populations studded, sample size, methods of data collection or method of assay for defining glycaemic control.

The higher rate of poor glycemic control observed among rural residents was consistent with the findings of previous study [19]. This might be due to the lower awareness, treatment and control of diabetes among persons living in rural areas [27]. Our finding in terms of level of education was also supported by other previous studies [20, 28, 29]. Low literacy is common in diabetic patients and is associated with poor glycaemic control [30, 31]. The incorporation of health literacy in diabetes care is therefore important as it has a significant impact on improvement of glycemic control [32]. Poor glycemic control appeared to be higher among merchants than government employees and this might be due to low literacy status of merchants in this study.

The lack of association between type of diabetes and glycemic control is not consistent with previous studies [19, 26]. It seems that type of diabetes is not important and what is important is the duration of diabetes. The worsening of glycaemic control over time has also been reported in other studies [19, 26, 28, 33] and could be explained by the progressive impairment of insulin secretion as a result of β-cell failure [8]. The poor glycemic control observed among patients receiving oral anti-diabetics or insulin was also supported by previous studies [34, 35]. However, studies have shown that the use of insulin or a combination of insulin and oral anti-diabetics are associated with poor glycemic control [20, 36]. Insulin therapy alone in type 2 diabetic patients has also shown to be associated with persistent poor glycemic control [19, 28].

### Conclusions

In conclusion, more than 70% of diabetic adults attending our clinic in Northeast Ethiopia had poor glycaemic control. These findings highlight the need for appropriate management of patients focusing on the associated factors identified for poor glycemic control to maintain good glycemic control.

## Limitations

The use of FBG over HbA1c is one limitation since a standardized method for measuring HbA1c was not available, thus possibly leading to underestimation of the prevalence of poor glycemic control. The cross-sectional nature of the study is other limitation, where better relationship between glycemic control and different potential factors affecting it progressively cannot be well established. The subjective nature of the self-reported response for some items might also be limited by recall bias.

## Abbreviations

AOR: adjusted odds ratio
BMI: body mass index
CI: confidence interval
DM: diabetes mellitus
DRH: Dessie Referral Hospital
FBG: fasting blood glucose
